# The effect of insulin on 28-day mortality in non-diabetic sepsis patients: a retrospective study

**DOI:** 10.3389/fcimb.2026.1806847

**Published:** 2026-04-15

**Authors:** Yifei Liu, Jie Yue, Nana Liu, Yuxia Jiang, Guangdong Wang, Aihua Shu

**Affiliations:** 1 Department of Anesthesiology, The First College of Clinical Medical Science, China Three Gorges University, Yichang, Hubei, China; 2 Yichang Central People’s Hospital, Yichang, Hubei, China; 3 The Institute of Geriatric Anesthesia, China Three Gorges University, Yichang, Hubei, China; 4 Department of Respiratory and Critical Care Medicine, First Affiliated Hospital of Xi’an Jiao tong University, Xi’an, Shanxi, China

**Keywords:** 28-day mortality, insulin, MIMIC-IV database, propensity score matching, sepsis

## Abstract

**Objective:**

To evaluate the association between insulin administration and 28-day mortality in non-diabetic patients with sepsis.

**Methods:**

A retrospective analysis of 11,289 critically ill patients with sepsis in the MIMIC-IV database was conducted. Propensity score matching (PSM) was employed to balance baseline characteristics between the insulin-treated and untreated cohorts. The primary outcome was 28-day mortality; secondary outcome measures included intensive care unit (ICU) and hospital length of stay (LOS). The association between insulin use and mortality was evaluated via Cox regression analysis, and survival was estimated using Kaplan-Meier curves and log-rank tests.

**Results:**

Of the 11,289 non-diabetic patients meeting the inclusion criteria, 1,172 (10.4%) received insulin. After PSM, the final analysis included 2,960 patients (782 in the insulin group and 2,178 in the control group). Baseline characteristics were well-balanced, with standardized mean differences (SMDs) close to zero. Insulin use was significantly associated with a reduced 28-day mortality rate (hazard ratio [HR] 0.63-0.67, p < 0.001), a finding that remained consistent across all subgroups. However, patients receiving insulin therapy had a longer median ICU LOS (5 days vs. 4 days, p < 0.001) and a more extended hospital LOS (12 days vs. 10 days, p < 0.001).

**Conclusion:**

Insulin therapy may improve the survival rate among non-diabetic patients with sepsis but does not shorten the duration of hospitalization. Further research is warranted to elucidate the underlying mechanism and validate these findings.

## 1 Introduction

Sepsis is a life-threatening organ dysfunction precipitated by a dysregulated host response to infection (Singer et al., 2016; van der Poll et al., 2021). It remains the leading cause of mortality in ICUs worldwide (Rello et al., 2017; Huang et al., 2019). The pathophysiology of sepsis is complex and biphasic. The initial phase is characterized by a pro-inflammatory “cytokine storm” involving a massive release of factors such as tumor necrosis factor-α (TNF-α), interleukin-1β (IL-1β), and IL-6, leading to microcirculatory dysfunction and tissue hypoxia (Chousterman et al., 2017; Minasyan, 2017). This is often followed by a state of immunoparalysis marked by immune cell apoptosis and an increased risk of secondary infections (Gao et al., 2025). Persistent mitochondrial dysfunction and excessive production of reactive oxygen species (ROS) throughout this process contribute to cellular energy exhaustion and organ failure (Prauchner, 2017; Nagar et al., 2018; Ow et al., 2021).

While insulin therapy has long been established as a standard of care to manage stress-induced hyperglycemia in critically ill patients (Bosch et al., 2021), recent evidence suggests that insulin may exert additional therapeutic effects beyond glycemic control (Polat et al., 2017; Casagrande et al., 2020). It can promote the clearance of pathogen-associated molecular patterns like lipopolysaccharides (LPS) via lipid metabolism regulation (Polat et al., 2017). Insulin may also improve mitochondrial respiration and enhance ATP generation by activating the AMPK pathway, thereby mitigating cellular energy exhaustion (Vanhorebeek et al., 2005; Rena et al., 2017). Furthermore, insulin plays a crucial role in immune-metabolic reprogramming; it augments glucose uptake via the PI3K/Akt pathway and modulates inflammatory responses through the MAPK pathway, helping to maintain the bactericidal activity of immune cells (Huang et al., 2018; Casagrande et al., 2020). Additionally, insulin has been shown to directly inhibit the activation of the NLRP3 inflammasome, reducing levels of pro-inflammatory cytokines like IL-6 and TNF-α (Andersen et al., 2004; Chang et al., 2021; Mehdi et al., 2025). These mechanisms suggest that insulin’s role in sepsis may extend beyond simply correcting hyperglycemia.

Given these potential pleiotropic effects, the present study aims to explore the association between insulin administration and mortality in non-diabetic patients with sepsis. By focusing on a non-diabetic cohort, we strive to eliminate the potential interference of pre-existing diabetes on insulin requirements and prior chronic exposure. Furthermore, we evaluate the impact of insulin on the secondary outcomes of ICU and hospital LOS. By employing PSM and multivariate analysis, we aim to provide robust evidence on the potential benefits of insulin therapy in managing sepsis.

## 2 Methods

### 2.1 Source of the data

This retrospective analysis was conducted using the MIMIC-IV database. Data extraction was performed by a researcher (SW; Certification Number: 11,301,845). The study adhered to the guidelines outlined in the Transparent Reporting of a Multivariable Prediction Model for Individual Prognosis or Diagnosis (TRIPOD) statement.

### 2.2 Study population

The inclusion criteria were as follows: patients admitted to the ICU for the first time during their index hospitalization, a stay exceeding 24 hours, and diagnostic criteria for sepsis. Patients with incomplete medical records or a history of type 1 or type 2 diabetes were excluded.

### 2.3 Data collection

Demographic characteristics extracted for analysis included age, gender, weight, and race. Clinical data encompassed vital signs (including heart rate (HR), respiratory rate (RR), systolic blood pressure peripheral capillary oxygen saturation [SpO_2_], and body temperature), acuity scores (Sequential Organ Failure Assessment [SOFA], APSIII, SAPSII, OASIS, Glasgow Coma Scale [GCS], Charlson Comorbidity Index, APACHE II, and SIRS), comorbidities (hypertension, acute kidney injury, cirrhosis, cerebrovascular accident, heart failure, myocardial infarction, ischemic heart disease, and chronic obstructive pulmonary disease), and biochemical indicators such as hemoglobin, platelets, red blood cells, white blood cells, blood glucose, creatinine, and lactate.

### 2.4 Exposure and outcomes of this study

Exposure was defined as the administration of insulin at any point during the patient’s ICU or hospital stay. Insulin use was specifically defined as the administration of short-acting (regular) insulin, which in this ICU cohort was predominantly delivered via continuous intravenous infusion for glycemic management. While the specific glycemic thresholds that prompted insulin initiation are not available in the database, its use in this context can be primarily interpreted as the clinical management of stress-induced hyperglycemia. This interpretation is strongly supported by the exclusion of all patients with pre-existing type 1 or type 2 diabetes from the study cohort, ensuring that insulin use was not a continuation of chronic outpatient therapy. The primary outcome was 28-day mortality, and the secondary outcomes were the number of days in the intensive care unit and total hospital stay.

### 2.5 Propensity score matching analysis

PSM was utilized to adjust for covariates to ensure the robustness of our findings. Variables included in the model were age, race, gender, comorbidities, and clinical scores, among others. SMDs were calculated to evaluate the effectiveness of the PSM.

### 2.6 Statistical analysis

Categorical variables were expressed as frequencies and percentages to describe the baseline distribution and group composition. For continuous variables, the mean and standard deviation were calculated to describe the central tendency and dispersion. Continuous data with a normal distribution were expressed as mean ± SD, while non-normally distributed data were expressed as median (interquartile range). Pearson chi-square tests were used to compare group differences for categorical variables. For continuous variables, independent t-tests and Wilcoxon rank-sum tests were selectively employed to obtain p-values, determining whether these variables exhibit statistical differences between the two groups, thereby analyzing whether the use of insulin is associated with changes in these variables as appropriate. Univariate Cox regression analysis was utilized to screen baseline variables; variables with P < 0.05 or other clinically relevant variables were incorporated into a multivariate Cox regression model. This yielded adjusted hazard ratios (HR), 95% confidence intervals (CI), and p-values, to clarify the independent effect of insulin on patients’ survival outcomes. Kaplan-Meier survival curves were used to estimate survival probability over time, followed by a log-rank test to assess group differences. Subgroup analyses were conducted using Cox multivariate regression, stratified by relevant clinical variables. All statistical analyses were performed using R software (v.4.2.0, R Foundation for Statistical Computing). A p-value < 0.05 was considered statistically significant.

## 3 Results

### 3.1 Baseline characteristics

**Figure 1:**
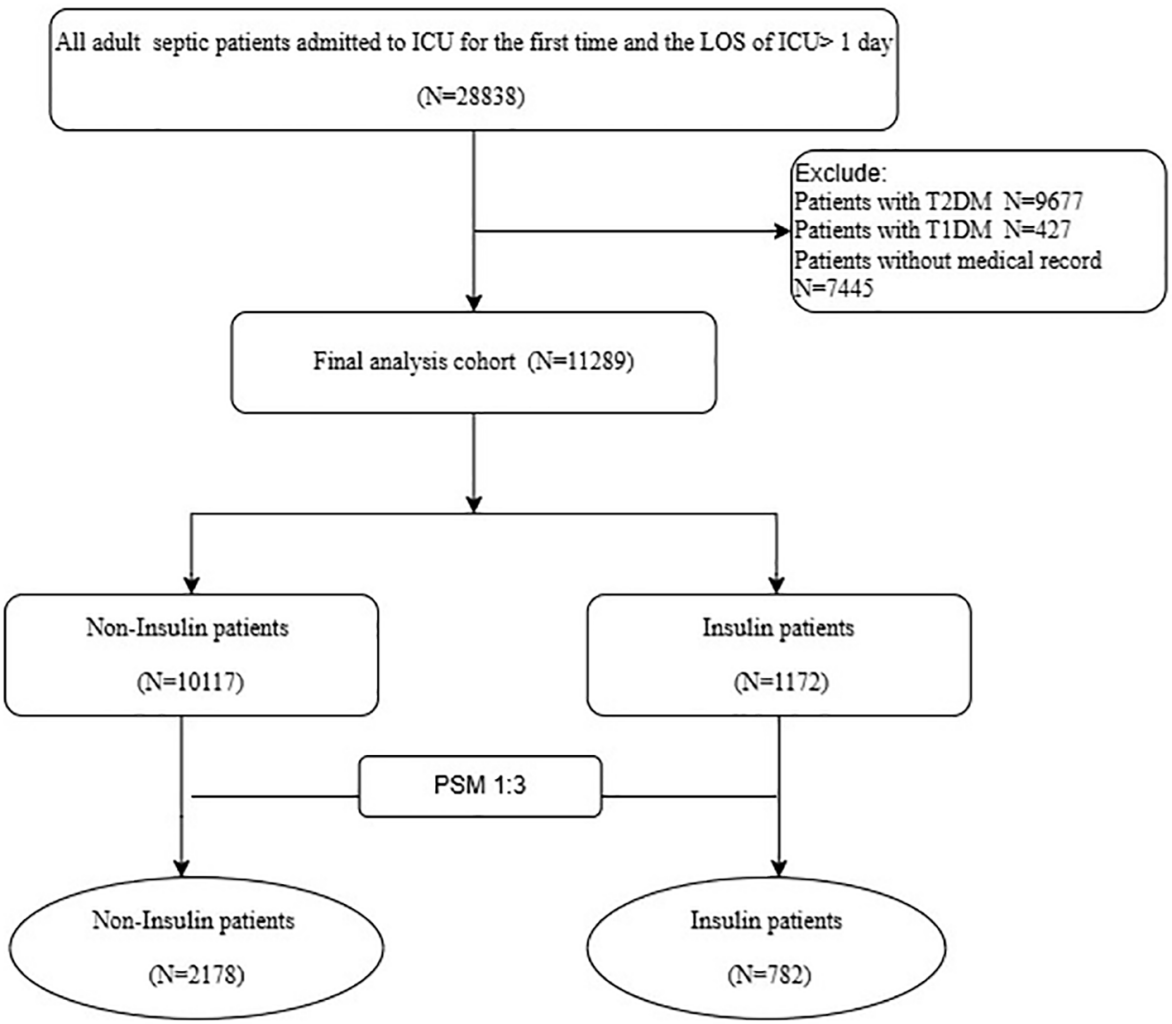
Study design flowchart.

**Figure 2:**
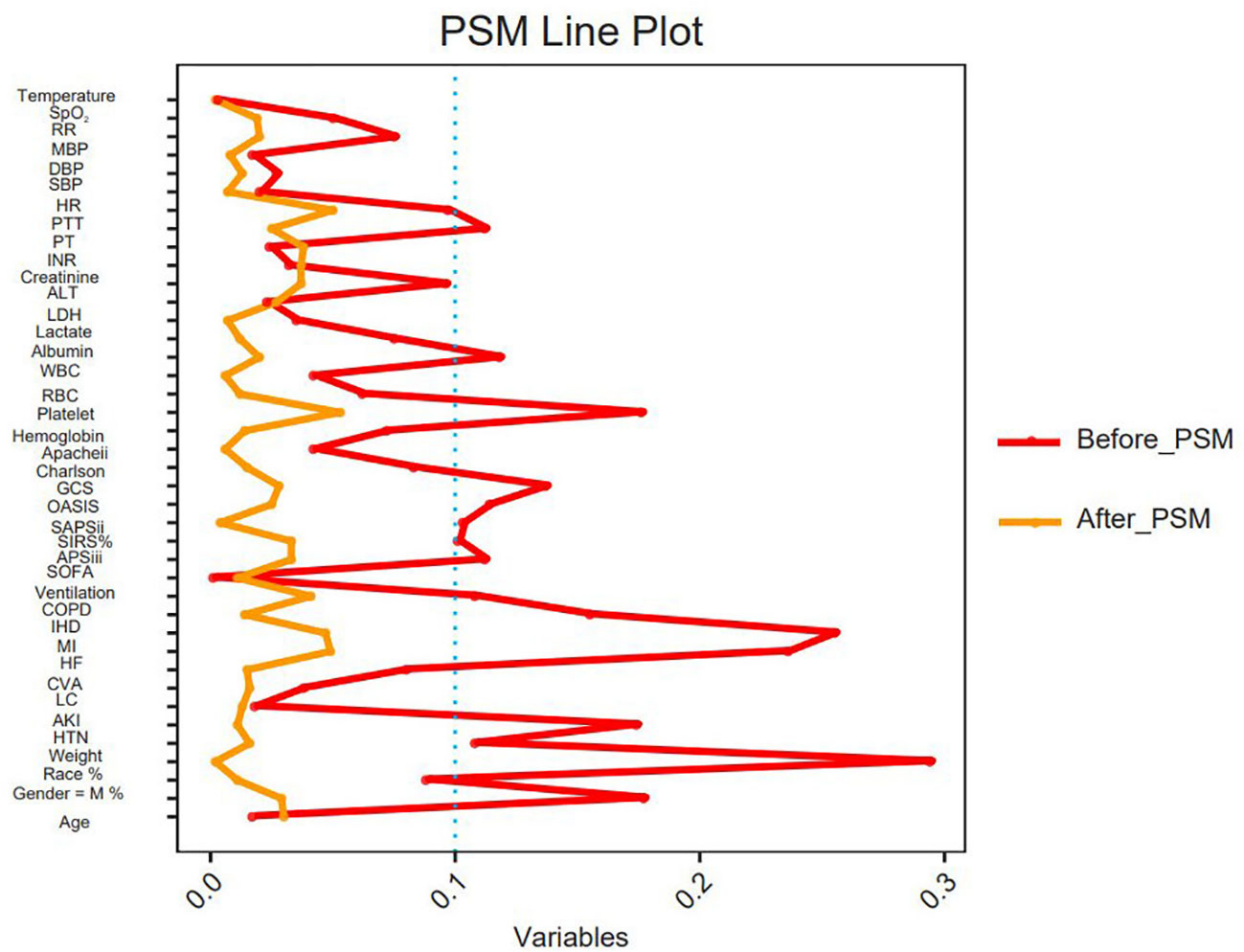
PSM line plot.

A total of 11,289 non-diabetic sepsis patients were included in this study, comprising 1,172 who received insulin therapy and 10,117 who did not. After PSM, a total of 2,960 patients were included (insulin group: 782; non-insulin group: 2,178) (**Figure 1**). Before matching, significant differences were observed between the two groups across several variables (including age, gender, race, weight, severity-of-illness scores, etc.), indicating baseline imbalance. After matching, the baseline characteristics of the two groups became well balanced, providing a reliable basis for subsequent analysis (**Table 1**). The PSM Line plot showed that SMDs for most key variables were reduced to near zero, indicating the successful mitigation of confounding bias (**Figure 2**). In both groups, patients in the nonsurvivor group tended to be older, exhibited higher severity scores (e.g., SOFA, APACHE II), and demonstrated worse laboratory parameters (e.g., elevated lactate and creatinine). These results suggest that advanced age, severe physiological derangement, and organ dysfunction are significant predictors of poor prognosis (**Tables 2**, **3**).

**Table 1:** Baseline characteristics of the non-insulin and insulin groups before and after PSM matching.

Variable	Group				Group			
	Non-insulin	Insulin	p	SMD	Non-insulin	Insulin	p-value	SMD
N	10117	1172			2178	782		
Age (years)	67 (18-100)	66 (20-92)	0.070	0.059	64 (18-99)	65 (20-92)	0.481	0.030
Gender (%)								
F	4367 (43.16)	426 (36.35)	<0.001	0.140	793 (36.41)	274 (35.04)	0.521	0.029
M	5750 (56.84)	746 (63.65)			1385 (63.59)	508 (64.96)		
Race (%)								
Black	701 (6.93)	92 (7.85)	0.011	0.092	172 (7.90)	64 (8.18)	0.964	0.011
Other	2856 (28.23)	372 (31.74)			700 (32.14)	252 (32.23)		
White	6560 (64.84)	708 (60.41)			1306 (59.96)	466 (59.59)		
Weight (Kg)	77.1 (1-390.7)	84.4 (29.2-211.8)	<0.001	0.282	82.642 (33-288.4)	84.7 (36-211.8)	0.970	0.002
SOFA	6 (0-22)	6 (0-21)	0.227	0.038	7 (0-21)	7 (0-20)	0.796	0.011
APSIII	49 (9-184)	47 (7-149)	0.001	0.107	50 (11-178)	50 (7-149)	0.433	0.033
SAPSII	40 (6-114)	39 (8-106)	0.006	0.087	39 (6-108)	39 (10-106)	0.931	0.004
OASIS	35 (6-68)	34 (11-59)	<0.001	0.116	34 (7-68)	34 (13-59)	0.545	0.025
GCS	15 (3-15)	15 (3-15)	<0.001	0.116	15 (3-15)	15 (3-15)	0.502	0.028
Charlson	5 (0-17)	4 (0-16)	0.001	0.109	4 (0-17)	4 (0-15)	0.720	0.015
APACHEII	19 (1-53)	20 (4-46)	0.015	0.077	20 (2-53)	21 (4-46)	0.894	0.006
SIRS	3 (0-4)	3 (0-4)	0.300	0.032	3 (1-4)	3 (1-4)	0.443	0.032
HR (bpm)	91 (0-191)	86 (0-941)	0.002	0.080	92 (0-182)	90 (0-179)	0.227	0.050
RR (bpm)	20 (0-91)	18 (0-50)	<0.001	0.130	20 (0-55)	19 (0-50)	0.628	0.020
SBP (mmHg)	117 (0-253)	116 (51-238)	0.234	0.038	117 (38-229)	116 (60-238)	0.869	0.007
DBP (mmHg)	67 (0-67168)	65 (11-156)	0.660	0.018	67 (11-176)	66 (18-156)	0.757	0.013
MBP (mmHg)	79 (0-6116)	77 (17-165)	0.379	0.036	79 (9-188)	78 (17-165)	0.854	0.008
SpO_2_ (%)	98 (0-963)	98 (70-9819)	0.002	0.043	98 (50-100)	98 (75-100)	0.650	0.019
Temperature (°F)	98.2 (0-106)	98.3 (34.1-103.6)	0.347	0.034	98.3 (0-106)	98.3 (34.1-103.2)	0.972	0.002
Hemoglobin (g/L)	10.6 (1.7-20)	10.2 (4-17.4)	<0.001	0.125	10.2 (2.8-19.8)	10.2 (4.5-17.4)	0.745	0.014
Platelet (10^9^/L)	182 (5-1647)	156 (6-909)	<0.001	0.236	163 (5-1647)	157 (6-909)	0.211	0.053
RBC (10^9^/L)	3.51 (0.59-7.25)	3.37 (1.15-6.34)	<0.001	0.115	3.39 (0.79-7.03)	3.36 (1.15-6.34)	0.764	0.012
WBC (10^9^/L)	11.8 (0.1-328.4)	12.3 (0.1-267.2)	0.172	0.041	12.2 (0.1-328.4)	12.4 (0.1-267.2)	0.893	0.006
Albumin (g/L)	2.9 (0.6-5.4)	3 (1-4.9)	0.011	0.085	3 (1-5.2)	3 (1-4.9)	0.629	0.020
Glucose (mmol/L)	124 (3-2044)	137 (42-1638)	<0.001	0.352	125 (3-817)	139 (42-1638)	<0.001	0.322
Lactate (mmol/L)	1.8 (0.2-29.2)	2 (0.5-19)	0.076	0.058	1.9 (0.2-21)	2 (0.5-19)	0.788	0.012
LDH (u/L)	301 (35-25010)	359.5 (97-16560)	0.557	0.020	329 (35-19980)	364 (103-16560)	0.865	0.007
ALT (u/L)	31 (0-13220)	31 (3-15018)	0.558	0.019	36 (2-9810)	33 (3-15018)	0.509	0.027
Creatinine (u/L)	1 (0-24.6)	1 (0.2-13.2)	0.004	0.096	1.1 (0.2-10.6)	1 (0.2-13.2)	0.367	0.037
INR	1.3 (0.8-21.1)	1.4 (0.9-12.3)	0.345	0.032	1.4 (0.8-13.8)	1.4 (0.9-12.3)	0.379	0.037
PT (s)	14.6 (8.3-150)	15.1 (9.4-150)	0.617	0.016	15 (8.3-150)	15.1 (9.9-130.9)	0.375	0.038
PTT (s)	31.7 (17.4-150)	32.1 (18.4-150)	0.001	0.096	32.45 (17.4-150)	32.4 (18.4-150)	0.553	0.025
HTN, n (p%)								
0	6272 (61.99)	671 (57.25)	0.002	0.097	1304 (59.87)	462 (59.08)	0.730	0.016
1	3845 (38.01)	501 (42.75)			874 (40.13)	320 (40.92)		
AKI, n (p%)								
0	5323 (52.61)	544 (46.42)	<0.001	0.124	853 (39.16)	302 (38.62)	0.822	0.011
1	4794 (47.39)	628 (53.58)			1325 (60.84)	480 (61.38)		
LC, n (p%)								
0	8960 (88.56)	1035 (88.31)	0.834	0.008	1862 (85.49)	672 (85.93)	0.808	0.013
1	1157 (11.44)	137 (11.69)			316 (14.51)	110 (14.07)		
CVA, n (p%)								
0	9294 (91.87)	1074 (91.64)	0.832	0.008	2009 (92.24)	718 (91.82)	0.763	0.016
1	823 (8.13)	98 (8.36)			169 (7.76)	64 (8.18)		
HF, n (p%)								
0	7261 (71.77)	812 (69.28)	0.080	0.055	1527 (70.11)	543 (69.44)	0.759	0.015
1	2856 (28.23)	360 (30.72)			651 (29.89)	239 (30.56)		
MI, n (p%)								
0	9284 (91.77)	990 (84.47)	<0.001	0.227	1860 (85.40)	654 (83.63)	0.260	0.049
1	833 (8.23)	182 (15.53)			318 (14.60)	128 (16.37)		
IHD, n (p%)								
0	7006 (69.25)	666 (56.83)	<0.001	0.260	1359 (62.40)	470 (60.10)	0.276	0.047
1	3111 (30.75)	506 (43.17)			819 (37.60)	312 (39.90)		
COPD, n (p%)								
0	8438 (83.40)	1042 (88.91)	<0.001	0.160	1926 (88.43)	695 (88.87)	0.787	0.014
1	1679 (16.60)	130 (11.09)			252 (11.57)	87 (11.13)		
Ventilation, n (p%)								
0	639 (6.32)	48 (4.10)	0.003	0.100	117 (5.37)	35 (4.48)	0.379	0.041
1	9478 (93.68)	1124 (95.90)			2061 (94.63)	747 (95.52)		
Vasopressin, n (p%)								
0	8535 (84.36)	920 (78.50)	0.143	<0.001	1785 (81.96)	590 (75.45)	0.151	<0.001
1	1582 (15.64)	252 (21.50)			393 (18.04)	192 (24.55)		

ApacheII, Acute Physiologic And Chronic Health Evaluation; APS III, Acute Physiology Score III; OASIS, Oxford Acute Severity of Illness Score; SAPSII, Scale for Assessment of Positive Symptoms; SIRS, Systemic Inflammatory Response Syndrome; SOFA, Sequential Organ Failure Assessment; INR, International Normalized Ratio; LDH, Lactate Dehydrogenase; SBP, Systolic Blood Pressure; PT, Prothrombin Time; PTT, Partial Thromboplastin Time; LC, Liver Cirrhosis; MI, Myocardial Infarction; COPD, Chronic Obstructive Pulmonary Disease; WBC, White blood cell count; RBC, Red blood cell count; AKI, Acute Kidney Injury; RR, Respiratory rate; HR, Heart Rate; SpO_2_, Oxygen saturation; Platelet, Platelet count.

**Table 2:** Hospitalized patient demographics and baseline characteristics.

Variable	Overall	Group		
		Hospitalized survival for more than 28 days	Hospitalized death within 28 days	p-value
	N = 2,960	N = 2,323	N = 637	
Insulin, n (p%)				<0.001
0	2,178.00 (73.58%)	1,672.00 (71.98%)	506.00 (79.43%)	
1	782.00 (26.42%)	651.00 (28.02%)	131.00 (20.57%)	
Age (years)	62.80 (16.60)	61.71 (16.58)	66.76 (16.09)	<0.001
Gender, n (p%)				0.552
F	1,067.00 (36.05%)	831.00 (35.77%)	236.00 (37.05%)	
M	1,893.00 (63.95%)	1,492.00 (64.23%)	401.00 (62.95%)	
Race, n (p%)				0.076
Black	236.00 (7.97%)	183.00 (7.88%)	53.00 (8.32%)	
Other	952.00 (32.16%)	725.00 (31.21%)	227.00 (35.64%)	
White	1,772.00 (59.86%)	1,415.00 (60.91%)	357.00 (56.04%)	
Weight (Kg)	87.23 (25.85)	87.13 (25.10)	87.59 (28.42)	0.712
SOFA	7.07 (3.84)	6.69 (3.60)	8.42 (4.34)	<0.001
ApSIII	54.28 (22.48)	50.99 (20.55)	66.25 (25.03)	<0.001
SAPSII	40.96 (14.64)	38.98 (13.68)	48.17 (15.75)	<0.001
OASIS	34.70 (8.48)	33.91 (8.15)	37.55 (9.03)	<0.001
Charlson	4.55 (2.70)	4.28 (2.63)	5.51 (2.74)	<0.001
APACHEII	20.93 (7.56)	20.04 (7.25)	24.21 (7.77)	<0.001
SIRS	2.93 (0.83)	2.91 (0.84)	3.01 (0.77)	0.002
GCS	13.57 (3.02)	13.62 (2.97)	13.39 (3.21)	0.104
HR (bpm)	93.41 (21.47)	92.83 (21.17)	95.53 (22.42)	0.007
RR (bpm)	20.28 (6.86)	19.88 (6.82)	21.74 (6.79)	<0.001
SBP (mmHg)	118.61 (24.52)	119.18 (24.51)	116.54 (24.43)	0.016
DBP (mmHg)	68.74 (18.95)	68.92 (18.96)	68.11 (18.93)	0.338
MBP (mmHg)	81.23 (19.29)	81.49 (19.36)	80.31 (19.03)	0.167
SpO_2_ (%)	96.43 (4.42)	96.60 (4.46)	95.82 (4.20)	<0.001
Temperature (°F)	98.17 (4.05)	98.19 (4.09)	98.11 (3.89)	0.664
Hemoglobin (g/L)	10.41 (2.53)	10.48 (2.50)	10.17 (2.59)	0.007
Platelet (10^9^/L)	181.03 (113.21)	181.36 (106.53)	179.81 (134.86)	0.788
RBC (10^9^/L)	3.46 (0.88)	3.48 (0.87)	3.36 (0.92)	0.004
WBC (10^9^/L)	14.60 (15.64)	14.08 (14.30)	16.51 (19.68)	0.004
Albumin (g/L)	2.98 (0.64)	3.01 (0.62)	2.84 (0.67)	<0.001
Glucose (mmol/L)	146.18 (77.99)	144.08 (78.01)	153.84 (77.49)	0.005
Lactate (mmol/L)	2.68 (2.36)	2.52 (2.08)	3.27 (3.10)	<0.001
LDH (u/L)	629.48 (1,227.45)	551.45 (1,060.54)	914.04 (1,673.30)	<0.001
ALT (u/L)	177.30 (651.06)	170.59 (646.69)	201.78 (666.69)	0.293
Creatinine (u/L)	1.47 (1.20)	1.42 (1.19)	1.67 (1.25)	<0.001
INR	1.62 (0.88)	1.57 (0.83)	1.81 (1.03)	<0.001
PT (s)	17.73 (9.52)	17.19 (8.96)	19.69 (11.09)	<0.001
PTT (s)	42.08 (27.36)	40.96 (26.32)	46.15 (30.55)	<0.001
HTN, n (p%)				0.056
0	1,766.00 (59.66%)	1,365.00 (58.76%)	401.00 (62.95%)	
1	1,194.00 (40.34%)	958.00 (41.24%)	236.00 (37.05%)	
AKI, n (p%)				<0.001
0	1,155.00 (39.02%)	1,023.00 (44.04%)	132.00 (20.72%)	
1	1,805.00 (60.98%)	1,300.00 (55.96%)	505.00 (79.28%)	
LC, n (p%)				<0.001
0	2,534.00 (85.61%)	2,025.00 (87.17%)	509.00 (79.91%)	
1	426.00 (14.39%)	298.00 (12.83%)	128.00 (20.09%)	
CVA, n (p%)				0.308
0	2,727.00 (92.13%)	2,134.00 (91.86%)	593.00 (93.09%)	
1	233.00 (7.87%)	189.00 (8.14%)	44.00 (6.91%)	
HF, n (p%)				0.224
0	2,070.00 (69.93%)	1,637.00 (70.47%)	433.00 (67.97%)	
1	890.00 (30.07%)	686.00 (29.53%)	204.00 (32.03%)	
MI, n (p%)				0.009
0	2,514.00 (84.93%)	1,994.00 (85.84%)	520.00 (81.63%)	
1	446.00 (15.07%)	329.00 (14.16%)	117.00 (18.37%)	
IHD, n (p%)				0.740
0	1,829.00 (61.79%)	1,439.00 (61.95%)	390.00 (61.22%)	
1	1,131.00 (38.21%)	884.00 (38.05%)	247.00 (38.78%)	
COPD, n (p%)				0.035
0	2,621.00 (88.55%)	2,072.00 (89.20%)	549.00 (86.19%)	
1	339.00 (11.45%)	251.00 (10.80%)	88.00 (13.81%)	
Ventilation, n (p%)				0.953
0	152.00 (5.14%)	119.00 (5.12%)	33.00 (5.18%)	
1	2,808.00 (94.86%)	2,204.00 (94.88%)	604.00 (94.82%)	

ApacheII, Acute Physiologic And Chronic Health Evaluation; APS III, Acute Physiology Score III; OASIS, Oxford Acute Severity of Illness Score; SAPSII, Scale for Assessment of Positive Symptoms; SIRS, Systemic Inflammatory Response Syndrome; SOFA, Sequential Organ Failure Assessment; INR, International Normalized Ratio; LDH, Lactate Dehydrogenase; SBP, Systolic Blood Pressure; PT, Prothrombin Time; PTT, Partial Thromboplastin Time; LC, Liver Cirrhosis; MI, Myocardial Infarction; COPD, Chronic Obstructive Pulmonary Disease; WBC, White blood cell count; RBC, Red blood cell count; AKI, Acute Kidney Injury; RR, Respiratory rate; HR, Heart Rate; SpO_2_, Oxygen saturation; Platelet, Platelet count.

**Table 3:** ICU patient demographics and baseline characteristics.

Variable	Overall	ICU survival for more than 28 days	ICU death within 28 days	p-value
	N = 2,960	N = 2,286	N = 674	
Insulin, n (p%)				0.003
0	2,178.00 (73.58%)	1,652.00 (72.27%)	526.00 (78.04%)	
1	782.00 (26.42%)	634.00 (27.73%)	148.00 (21.96%)	
Age (years)	62.80 (16.60)	61.69 (16.63)	66.55 (15.96)	<0.001
Weight (Kg)	87.23 (25.85)	87.30 (25.09)	86.98 (28.28)	0.789
Gender, n (p%)				0.359
F	1,067.00 (36.05%)	814.00 (35.61%)	253.00 (37.54%)	
M	1,893.00 (63.95%)	1,472.00 (64.39%)	421.00 (62.46%)	
Race, n (p%)				0.155
Black	236.00 (7.97%)	179.00 (7.83%)	57.00 (8.46%)	
Other	952.00 (32.16%)	717.00 (31.36%)	235.00 (34.87%)	
White	1,772.00 (59.86%)	1,390.00 (60.80%)	382.00 (56.68%)	
SOFA	7.07 (3.84)	6.67 (3.59)	8.43 (4.31)	<0.001
APSIII	54.28 (22.48)	50.70 (20.40)	66.39 (24.85)	<0.001
SAPSII	40.96 (14.64)	38.81 (13.61)	48.24 (15.67)	<0.001
OASIS	34.70 (8.48)	33.88 (8.13)	37.47 (9.01)	<0.001
GCS	NA (NA)	NA (NA)	NA (NA)	
Charlson	4.55 (2.70)	4.26 (2.63)	5.53 (2.72)	<0.001
APACHEII	20.93 (7.56)	19.96 (7.23)	24.25 (7.73)	<0.001
SIRS	2.93 (0.83)	2.90 (0.84)	3.01 (0.78)	0.001
HR (bpm)	93.41 (21.47)	92.59 (21.04)	96.19 (22.69)	<0.001
RR (bpm)	20.28 (6.86)	19.82 (6.79)	21.86 (6.87)	<0.001
SBP (mmHg)	118.61 (24.52)	119.17 (24.52)	116.71 (24.44)	0.022
DBP (mmHg)	68.74 (18.95)	68.93 (18.91)	68.12 (19.08)	0.333
MBP (mmHg)	81.23 (19.29)	81.50 (19.31)	80.33 (19.19)	0.164
SpO_2_ (%)	96.43 (4.42)	96.63 (4.44)	95.75 (4.28)	<0.001
Temperature (°F)	98.17 (4.05)	98.19 (4.12)	98.12 (3.80)	0.676
Hemoglobin (g/L)	10.41 (2.53)	10.51 (2.50)	10.07 (2.59)	<0.001
Platelet (10^9^/L)	181.03 (113.21)	181.92 (105.83)	177.98 (135.31)	0.487
RBC (10^9^/L)	3.46 (0.88)	3.49 (0.87)	3.33 (0.92)	<0.001
WBC (10^9^/L)	14.60 (15.64)	14.05 (13.67)	16.47 (20.90)	0.005
Albumin (g/L)	2.98 (0.64)	3.02 (0.62)	2.83 (0.67)	<0.001
Glucose (mmol/L)	146.18 (77.99)	144.00 (78.03)	153.56 (77.42)	0.005
Lactate (mmol/L)	2.68 (2.36)	2.51 (2.08)	3.24 (3.07)	<0.001
LDH (u/L)	629.48 (1,227.45)	552.39 (1,066.04)	890.93 (1,636.19)	<0.001
ALT (u/L)	177.30 (651.06)	172.64 (651.68)	193.09 (649.17)	0.473
Creatinine (u/L)	1.47 (1.20)	1.41 (1.17)	1.68 (1.29)	<0.001
INR	1.62 (0.88)	1.57 (0.83)	1.81 (1.02)	<0.001
PT (s)	17.73 (9.52)	17.13 (8.95)	19.74 (10.99)	<0.001
PTT (s)	42.08 (27.36)	40.80 (26.05)	46.42 (31.03)	<0.001
HTN, n (p%)				0.132
0	1,766.00 (59.66%)	1,347.00 (58.92%)	419.00 (62.17%)	
1	1,194.00 (40.34%)	939.00 (41.08%)	255.00 (37.83%)	
AKI, n (p%)				<0.001
0	1,155.00 (39.02%)	1,015.00 (44.40%)	140.00 (20.77%)	
1	1,805.00 (60.98%)	1,271.00 (55.60%)	534.00 (79.23%)	
LC, n (p%)				<0.001
0	2,534.00 (85.61%)	1,996.00 (87.31%)	538.00 (79.82%)	
1	426.00 (14.39%)	290.00 (12.69%)	136.00 (20.18%)	
CVA, n (p%)				0.251
0	2,727.00 (92.13%)	2,099.00 (91.82%)	628.00 (93.18%)	
1	233.00 (7.87%)	187.00 (8.18%)	46.00 (6.82%)	
HF, n (p%)				0.323
0	2,070.00 (69.93%)	1,609.00 (70.38%)	461.00 (68.40%)	
1	890.00 (30.07%)	677.00 (29.62%)	213.00 (31.60%)	
MI, n (p%)				0.017
0	2,514.00 (84.93%)	1,961.00 (85.78%)	553.00 (82.05%)	
1	446.00 (15.07%)	325.00 (14.22%)	121.00 (17.95%)	
IHD, n (p%)				0.895
0	1,829.00 (61.79%)	1,414.00 (61.85%)	415.00 (61.57%)	
1	1,131.00 (38.21%)	872.00 (38.15%)	259.00 (38.43%)	
COPD, n (p%)				0.042
0	2,621.00 (88.55%)	2,039.00 (89.20%)	582.00 (86.35%)	
1	339.00 (11.45%)	247.00 (10.80%)	92.00 (13.65%)	
Ventilation, n (p%)				0.938
0	152.00 (5.14%)	117.00 (5.12%)	35.00 (5.19%)	
1	2,808.00 (94.86%)	2,169.00 (94.88%)	639.00 (94.81%)	

ApacheII, Acute Physiologic And Chronic Health Evaluation; APS III, Acute Physiology Score III; OASIS, Oxford Acute Severity of Illness Score; SAPSII, Scale for Assessment of Positive Symptoms; SIRS, Systemic Inflammatory Response Syndrome; SOFA, Sequential Organ Failure Assessment; INR, International Normalized Ratio; LDH, Lactate Dehydrogenase; SBP, Systolic Blood Pressure; PT, Prothrombin Time; PTT, Partial Thromboplastin Time; LC, Liver Cirrhosis; MI, Myocardial Infarction; COPD, Chronic Obstructive Pulmonary Disease; WBC, White blood cell count; RBC, Red blood cell count; AKI, Acute Kidney Injury; RR, Respiratory rate; HR, Heart Rate; SpO_2_, Oxygen saturation; Platelet, Platelet count.

### 3.2 Univariate analysis

Univariate Cox regression analysis revealed that insulin use was significantly associated with a reduced risk of mortality. This protective effect was validated in both hospitalized (HR = 0.675) and ICU cohorts (HR = 0.738). Conversely, variables such as age ≥65 years, elevated severity-of-illness scores, and increased lactate levels were strongly associated with an increased risk of mortality. Notably, variables including gender, weight, and race did not significantly affect prognosis, thereby establishing a basis for variable selection in subsequent multivariate analysis (**Tables 4**, **5**).

**Table 4:** Single factor Cox analysis of 28-day hospitalization mortality.

Variable	Number (%)	Hazard ratio (HR)	Lower_95	Upper_95	p-value
Insulin n (p%)					
0	2178 (73.6)				
1	782 (26.4)	0.675	0.557	0.818	0
Age (years)					
<65	1481 (50.0)				
≥65	1479 (50.0)	1.52	1.298	1.78	0
Gender, n (p%)					
F	1067 (36.0)				
M	1893 (64.0)	0.96	0.817	1.127	0.615
Weight (Kg)	87.23 (25.85)	1.001	0.998	1.004	0.708
Race, n (p%)					
Black	236 (8.0)				
Other	952 (32.2)	1.072	0.795	1.445	0.649
White	1772 (59.9)	0.879	0.659	1.173	0.381
SOFA	7.07 (3.84)	1.11	1.089	1.131	0
APACHEII	20.93 (7.56)	1.065	1.054	1.075	0
APSIII	54.27 (22.48)	1.024	1.021	1.027	0
Charlson	4.55 (2.70)	1.148	1.118	1.178	0
GCS	13.57 (3.02)	0.979	0.955	1.003	0.081
OASIS	34.70 (8.48)	1.047	1.038	1.057	0
SAPSII	40.96 (14.64)	1.036	1.031	1.041	0
SIRS	2.93 (0.83)	1.162	1.057	1.279	0.002
HR (bpm)	93.41 (21.47)	1.006	1.002	1.009	0.003
SBP (mmHg)	118.61 (24.52)	0.996	0.993	0.999	0.019
DBP (mmHg)	68.74 (18.95)	0.998	0.994	1.002	0.391
MBP (mmHg)	81.23 (19.29)	0.997	0.993	1.001	0.198
RR (bpm)	20.28 (6.86)	1.033	1.022	1.044	0
SpO_2_ (%)	96.43 (4.42)	0.972	0.958	0.985	0
Temperature (°F)	98.17 (4.05)	0.996	0.979	1.013	0.627
ALT (u/L)	177.30 (651.06)	1	1	1	0.234
Albumin (g/L)	2.98 (0.64)	0.684	0.604	0.773	0
Creatinine (u/L)	1.47 (1.20)	1.128	1.073	1.185	0
Glucose (mmol/L)	146.18 (77.99)	1.001	1	1.002	0.003
Hemoglobin (g/L)	10.41 (2.53)	0.959	0.929	0.99	0.009
Lactate (mmol/L)	2.68 (2.36)	1.107	1.08	1.135	0
LDH (u/L)	629.48 (1227.45)	1	1	1	0
RBC (10^9^/L)	3.46 (0.88)	0.877	0.801	0.959	0.004
WBC (10^9^/L)	14.60 (15.64)	1.006	1.003	1.009	0
Platelet (10^9^/L)	181.03 (113.21)	1	0.999	1.001	0.757
INR	1.62 (0.88)	1.186	1.122	1.253	0
PT (s)	17.73 (9.52)	1.016	1.011	1.021	0
PTT (s)	42.08 (27.36)	1.005	1.003	1.008	0
AKI, n (p%)					
0	1155 (39.0)				
1	1805 (61.0)	2.667	2.202	3.23	0
COPD, n (p%)					
0	2621 (88.5)				
1	339 (11.5)	1.272	1.015	1.593	0.036
CVA, n (p%)					
0	2727 (92.1)				
1	233 (7.9)	0.855	0.629	1.161	0.316
HF, n (p%)					
0	2070 (69.9)				
1	890 (30.1)	1.105	0.936	1.305	0.239
HTN, n (p%)					
0	1766 (59.7)				
1	1194 (40.3)	0.855	0.728	1.004	0.055
IHD, n (p%)					
0	1829 (61.8)				
1	1131 (38.2)	1.039	0.886	1.218	0.642
LC, n (p%)					
0	2534 (85.6)				
1	426 (14.4)	1.581	1.302	1.919	0
MI, n (p%)					
0	2514 (84.9)				
1	446 (15.1)	1.331	1.089	1.626	0.005
Ventilation, n (p%)					
0	152 (5.1)				
1	2808 (94.9)	0.947	0.667	1.344	0.759

ApacheII, Acute Physiologic And Chronic Health Evaluation; APS III, Acute Physiology Score III; OASIS, Oxford Acute Severity of Illness Score; SAPSII, Scale for Assessment of Positive Symptoms; SIRS, Systemic Inflammatory Response Syndrome; SOFA, Sequential Organ Failure Assessment; INR, International Normalized Ratio; LDH, Lactate Dehydrogenase; SBP, Systolic Blood Pressure; PT, Prothrombin Time; PTT, Partial Thromboplastin Time; LC, Liver Cirrhosis; MI, Myocardial Infarction; COPD, Chronic Obstructive Pulmonary Disease; WBC, White blood cell count; RBC, Red blood cell count; AKI, Acute Kidney Injury; RR, Respiratory rate; HR, Heart Rate; SpO_2_, Oxygen saturation; Platelet, Platelet count.

**Table 5:** Single factor Cox analysis of 28-day ICU mortality.

Variable	Number (%)	Hazard ratio (HR)	Lower_95	Upper_95	p-value
Insulin n (p%)					
0	2178 (73.6)				
1	782 (26.4)	0.738	0.615	0.885	0.001
Age (years)					
<65	1481 (50.0)				
≥65	1479 (50.0)	1.494	1.282	1.742	0
Gender, n (p%)					
F	1067 (36.0)				
M	1893 (64.0)	0.931	0.797	1.088	0.371
Weight (Kg)	87.23 (25.85)	1	0.997	1.002	0.744
Race, n (p%)					
Black	236 (8.0)				
Other	952 (32.2)	1.031	0.772	1.377	0.838
White	1772 (59.9)	0.88	0.666	1.162	0.367
SOFA	7.07 (3.84)	1.113	1.092	1.134	0
APACHEII	20.93 (7.56)	1.066	1.057	1.076	0
APSIII	54.27 (22.48)	1.025	1.022	1.027	0
Charlson	4.55 (2.70)	1.152	1.123	1.182	0
GCS	13.57 (3.02)	0.981	0.958	1.004	0.107
OASIS	34.70 (8.48)	1.046	1.037	1.056	0
SAPSII	40.96 (14.64)	1.037	1.032	1.042	0
SIRS	2.93 (0.83)	1.168	1.065	1.282	0.001
HR (bpm)	93.41 (21.47)	1.007	1.004	1.011	0
RR (bpm)	20.28 (6.86)	1.036	1.026	1.047	0
SpO_2_ (%)	96.43 (4.42)	0.969	0.956	0.982	0
Temperature (°F)	98.17 (4.05)	0.996	0.98	1.013	0.662
SBP (mmHg)	118.61 (24.52)	0.996	0.993	0.999	0.022
DBP (mmHg)	68.74 (18.95)	0.998	0.994	1.002	0.366
MBP (mmHg)	81.23 (19.29)	0.997	0.993	1.001	0.18
ALT (u/L)	177.30 (651.06)	1	1	1	0.419
Albumin (g/L)	2.98 (0.64)	0.667	0.591	0.752	0
Creatinine (u/L)	1.47 (1.20)	1.131	1.078	1.186	0
Glucose (mmol/L)	146.18 (77.99)	1.001	1	1.002	0.003
Hemoglobin (g/L)	10.41 (2.53)	0.939	0.91	0.968	0
Lactate (mmol/L)	2.68 (2.36)	1.105	1.079	1.133	0
LDH (u/L)	629.48 (1227.45)	1	1	1	0
RBC (10^9^/L)	3.46 (0.88)	0.823	0.753	0.899	0
WBC (10^9^/L)	14.60 (15.64)	1.006	1.003	1.009	0
Platelet (10^9^/L)	181.03 (113.21)	1	0.999	1	0.395
INR	1.62 (0.88)	1.191	1.13	1.256	0
PT (s)	17.73 (9.52)	1.016	1.011	1.021	0
PTT (s)	42.08 (27.36)	1.006	1.003	1.008	0
AKI, n (p%)					
0	1155 (39.0)				
1	1805 (61.0)	2.681	2.226	3.23	0
COPD, n (p%)					
0	2621 (88.5)				
1	339 (11.5)	1.252	1.005	1.56	0.045
CVA, n (p%)					
0	2727 (92.1)				
1	233 (7.9)	0.849	0.629	1.145	0.284
HF, n (p%)					
0	2070 (69.9)				
1	890 (30.1)	1.082	0.92	1.273	0.342
HTN, n (p%)					
0	1766 (59.7)				
1	1194 (40.3)	0.884	0.756	1.033	0.12
IHD, n (p%)					
0	1829 (61.8)				
1	1131 (38.2)	1.021	0.874	1.192	0.797
LC, n (p%)					
0	2534 (85.6)				
1	426 (14.4)	1.617	1.339	1.951	0
MI, n (p%)					
0	2514 (84.9)				
1	446 (15.1)	1.298	1.066	1.58	0.009
Ventilation, n (p%)					
0	152 (5.1)				
1	2808 (94.9)	0.956	0.681	1.344	0.797

ApacheII, Acute Physiologic And Chronic Health Evaluation; APS III, Acute Physiology Score III; OASIS, Oxford Acute Severity of Illness Score; SAPSII, Scale for Assessment of Positive Symptoms; SIRS, Systemic Inflammatory Response Syndrome; SOFA, Sequential Organ Failure Assessment; INR, International Normalized Ratio; LDH, Lactate Dehydrogenase; SBP, Systolic Blood Pressure; PT, Prothrombin Time; PTT, Partial Thromboplastin Time; LC, Liver Cirrhosis; MI, Myocardial Infarction; COPD, Chronic Obstructive Pulmonary Disease; WBC, White blood cell count; RBC, Red blood cell count; AKI, Acute Kidney Injury; RR, Respiratory rate; HR, Heart Rate; SpO_2_, Oxygen saturation; Platelet, Platelet count.

### 3.3 Multifactorial analysis

The results of multivariate Cox regression analysis demonstrated that insulin use was associated with a lower risk of mortality both before and after matching (HR range: 0.63-0.67, p < 0.001). Even after adjusting for multiple confounding factors, including age, severity-of-illness scores, and laboratory indicators, the protective effect of insulin remained significant. These findings suggest that insulin administration independently improves survival outcomes in non-diabetic sepsis patients (**Table 6**).

**Table 6:** The association between insulin and all-cause mortality at 28 days mortality in sepsis.

Variables	Model 1		Model 2		Model 3	
	HR (95% CI)	*p-value*	HR (95% CI)	*p-value*	HR (95% CI)	*p-value*
After PSM 28-day						
Insulin	0.675 (0.557, 0.818)	<0.001	0.655 (0.54, 0.794)	<0.001	0.672 (0.514, 0.764)	<0.001
Before PSM 28-day						
Insulin	0.623 (0.536, 0.724)	<0.001	0.651 (0.56, 0.754)	<0.001	0.63 (0.529, 0.751)	<0.001

### 3.4 Survival analysis and subgroup analysis

**Figure 3:**
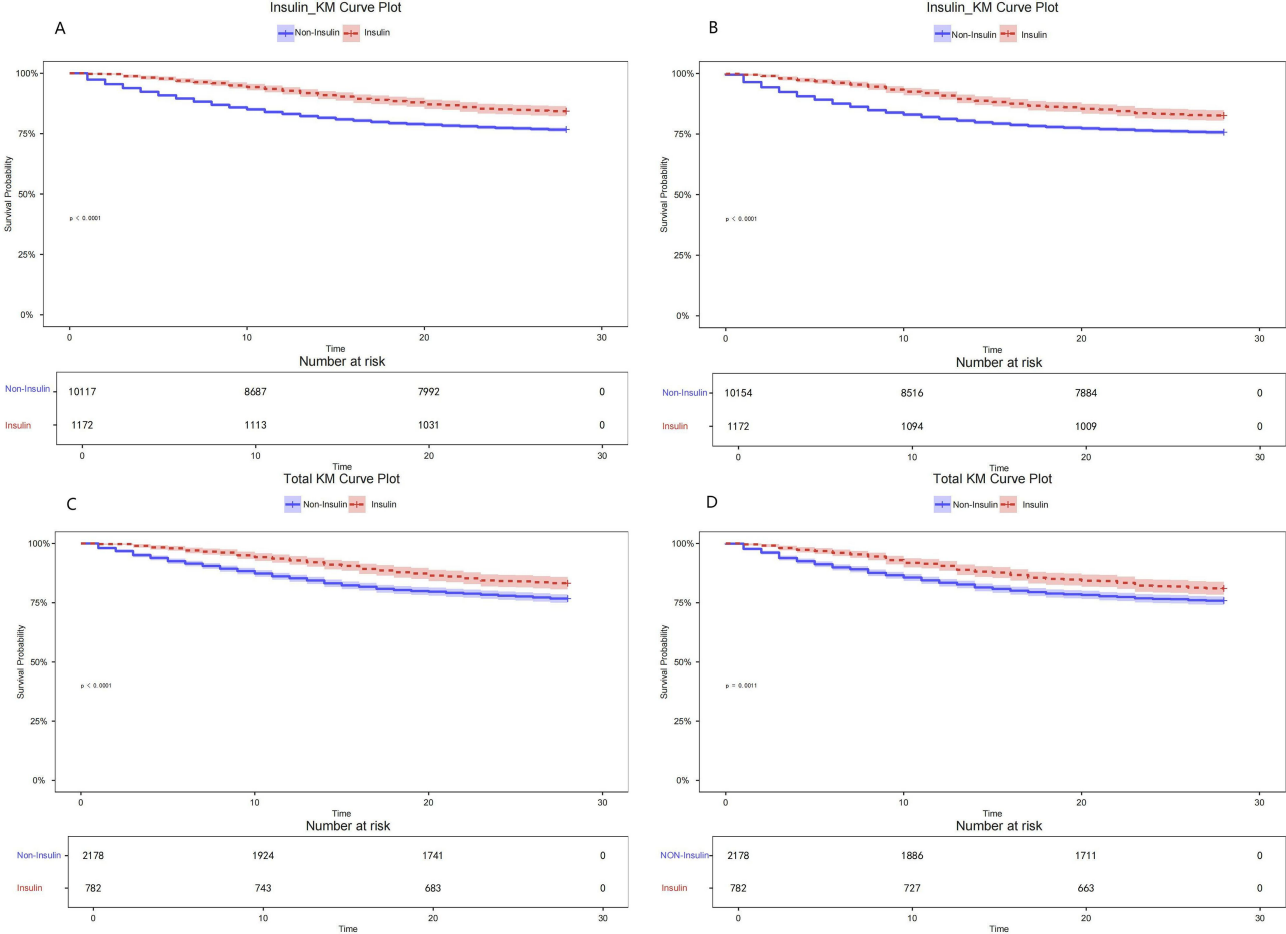
28-day survival analysis curves. **(A)** Hospitalized patients before PSM; **(B)** ICU patients before PSM; **(C)** Hospitalized patients after PSM; **(D)** ICU patients after PSM.

Survival curves for hospitalized and ICU patients demonstrated a distinct separation between the insulin group and the non-insulin group before matching, reflecting a significant difference in survival rates (p < 0.001). While confounding factors may have influenced this initial difference, the separation remained significant after PSM (p < 0.001). This persistence indicates that the association between insulin therapy and reduced mortality risk remains robust even after adjusting for baseline characteristics, further confirming that insulin administration exerts an independent protective effect against short-term mortality in the ICU setting (**Figure 3**).

**Figure 4:**
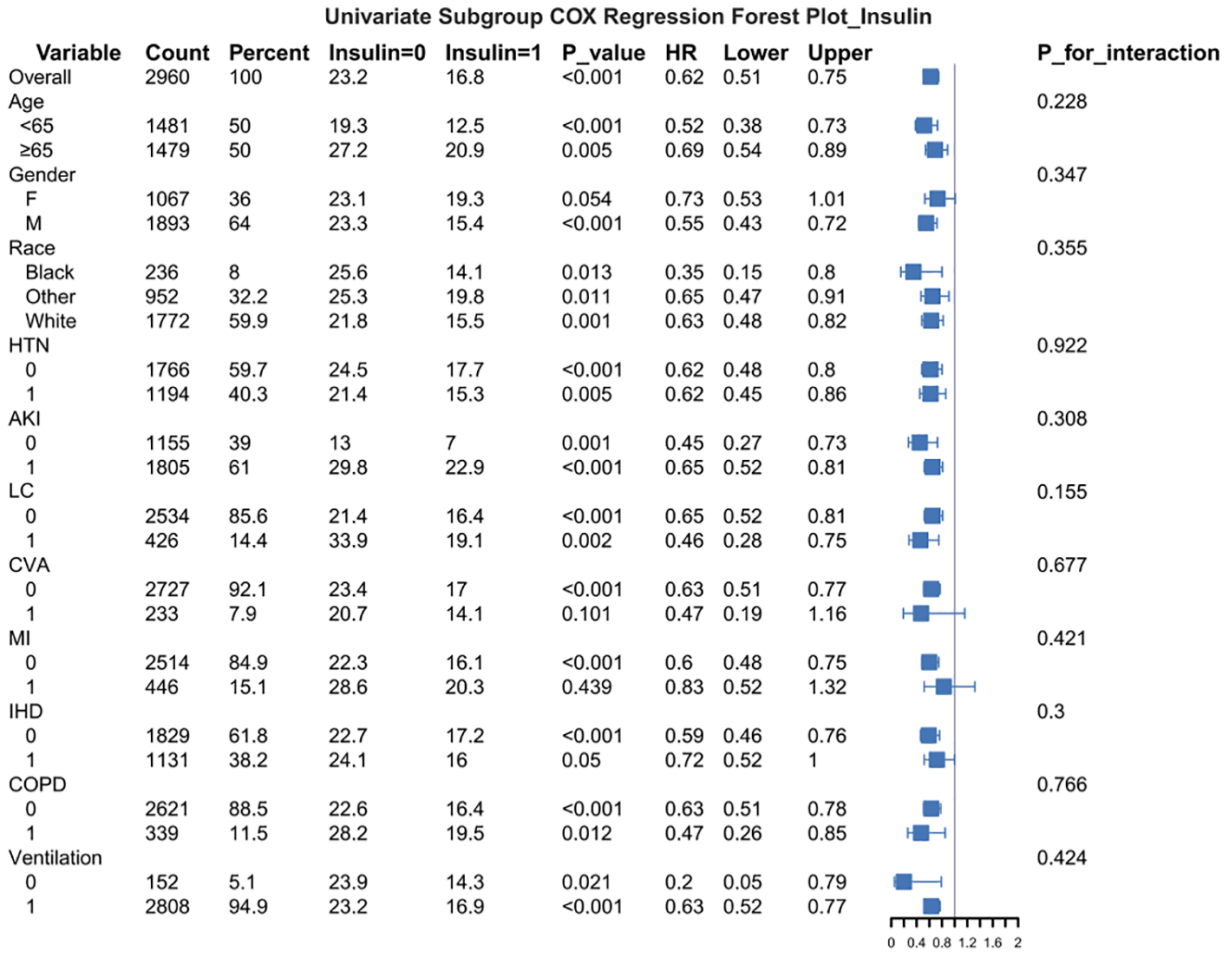
Univariate subgroup COX regression forest plot.

Subgroup analysis results indicated that insulin use was associated with a significantly reduced risk of death (HR = 0.62, p < 0.001). This protective effect was consistent across all analyzed strata, including age groups, gender, race, and patients with comorbidities. Although specific comorbidities (e.g., AKI, cirrhosis, and COPD) were associated with an increased baseline risk of death, insulin therapy consistently demonstrated a significant protective effect within these high-risk subgroups (**Figure 4**).

### 3.5 Secondary results

Consistent with the findings before and after matching, the ICU and hospital LOS in the insulin group were significantly longer than those in the control group (p < 0.001). This observation suggests that patients receiving insulin may have possessed more complex medical conditions requiring extended clinical management. Although insulin use was associated with a lower risk of mortality, its impact on healthcare resource utilization, such as hospital LOS, requires further investigation (**Table 7**).

**Table 7:** Treatment and secondary outcomes.

Variable	Before PSM			After PSM		
	Non-insulin	Insulin	p-value	Non-insulin	Insulin	p-value
	10117	1172		2178	782	
Events						
Los of ICU(days)	4.48 (1.01-159.67)	5.9 (1.01-140.05)	<0.001	5.095 (1.01-94.06)	7.39 (1.01-140.05)	<0.001
Los of Hospital (days)	11.66 (0.11-321.58)	16.045 (1-246.58)	<0.001	13.555 (1.02-206.43)	20.305 (1-246.58)	<0.001

## 4 Discussion

This study found that among non-diabetic sepsis patients, insulin administration was significantly associated with reduced 28-day mortality after propensity score matching, despite being linked to longer ICU and hospital stays. The protective effect of insulin remained robust across all analyses, suggesting an independent benefit independent of baseline characteristics.

### 4.1 Insulin improves the prognosis of septic patients

The core finding of this study—that insulin administration is significantly associated with a reduced 28-day mortality rate (multivariable adjusted HR = 0.63-0.67, p<0.001)—provides compelling real-world evidence supporting the application of intensive insulin therapy (IIT) in sepsis. This effect persisted after PSM, suggesting its independence from baseline confounding factors. Critically ill patients often develop stress-induced hyperglycemia, and current guidelines recommend initiating intravenous insulin when blood glucose remains ≥180 mg/dL (Bosch et al., 2021). Although the insulin group exhibited longer ICU and hospital LOS, which may reflect more complex underlying conditions (e.g., higher baseline SOFA scores) requiring extended recovery time, survival curve analysis clearly demonstrated a sustained survival benefit throughout the entire observation period.

The ability of insulin to exert this protective effect is particularly remarkable given the profound insulin resistance commonly observed in septic patients. As highlighted by Illuri et al., critically ill patients with sepsis can develop extreme insulin resistance, defined as an insulin requirement of >3 units/kg/day, which can reverse as rapidly as it appears (Illuri et al., 2016). This resistance is primarily driven by inflammatory cytokines and counter-regulatory hormones that impair insulin signaling. In this context, exogenously administered insulin may overcome this blockade through a concentration-dependent mechanism. Supraphysiologic doses can saturate remaining functional receptors or bypass proximal signaling defects to activate downstream pathways such as PI3K/Akt and AMPK, thereby restoring some degree of metabolic control and anti-inflammatory action despite the hostile cytokine environment. This mechanistic understanding supports our finding that insulin administration, even in severely ill patients, can be associated with a survival advantage.

Hyperglycemia exacerbates sepsis progression by activating the NF-κB pathway, impairing leukocyte chemotaxis and phagocytic function, and increasing vascular endothelial permeability (Andersen et al., 2004). The ability of insulin to reverse these effects suggests that metabolic support is as critical as hemodynamic stabilization. Acute hyperglycemia (AH) is a significant risk factor for sepsis-associated encephalopathy (SAE) and delirium, and insulin therapy may reverse hyperglycemia-induced neuroinflammation and cognitive impairment (Yao et al., 2024).

Furthermore, the observed survival benefit may extend beyond these acute anti-inflammatory effects. A growing body of evidence suggests that restoring normoglycemia itself confers long-term cardiometabolic protection. In the Diabetes Prevention Program Outcomes Study (DPPOS) and the Da Qing study, regression from prediabetes to normoglycemia was associated with a substantially reduced risk of cardiovascular death and heart failure over decades of follow-up (Vazquez Arreola et al., 2026). Although these findings are derived from outpatient populations, they provide compelling mechanistic plausibility for our results. They suggest that in septic patients, insulin-mediated reversal of stress hyperglycemia may initiate a beneficial metabolic legacy effect, reducing downstream cardiovascular complications and contributing to the 28-day mortality reduction observed in our cohort. This reframes the role of insulin not merely as a glucose-lowering agent but as a metabolic resuscitator.

In parallel with its effects on host metabolism, insulin may also directly influence pathogen behavior. Recent evidence by Plotkin et al. has established insulin as an interkingdom quorum-sensing molecule. They demonstrated that the insulin-to-glucose ratio over the physiologic range critically modulates biofilm formation in both Gram-positive and Gram-negative bacteria, including multi-drug resistant clinical isolates, with maximal biofilm levels observed at 220 mg/dL glucose (Plotkin et al., 2023). In sepsis, where biofilm formation on catheters or native valves can perpetuate infection, insulin therapy might inadvertently or beneficially alter bacterial behavior. By modulating the ratio of insulin to glucose, exogenous administration could potentially suppress biofilm formation or enhance planktonic phenotypes, rendering bacteria more susceptible to antibiotics and host immune clearance. This dual effect—modulating host immunity while directly influencing pathogen virulence—provides a novel paradigm for understanding the mortality benefit observed in our study and warrants further investigation into organism-specific responses to insulin therapy.

Notably, data from this study indicate that the protective effect of insulin is significant in non-diabetic sepsis patients, diverging from the previously emphasized ‘strict blood glucose control’ strategy. This suggests that its benefits stem from immune-metabolic regulation rather than pure normalization of blood glucose alone.

However, paradoxically, spontaneous (non-iatrogenic) hypoglycemia is significantly associated with mortality (OR = 1.65). In contrast, insulin-related hypoglycemia, if not severe enough to mask infection symptoms (such as coma), has a lower direct risk of death (Wang J. et al., 2021). At the same time, some studies suggest that individualized glycemic management strategies may improve sepsis outcomes, particularly in non-diabetic patients (Li et al., 2024). However, the NICE-SUGAR (The Normoglycemia in Intensive Care Evaluation-Survival Using Glucose Algorithm Regulation) study demonstrated that strict IIT increases the risk of hypoglycemia without significantly improving prognosis (Finfer et al., 2009; Krinsley, 2015; Rivas and Nugent, 2021). Our study did not find that insulin increases hypoglycemia-related mortality, though it cannot rule out the possibility of delayed recovery. Future research should clarify the impact of blood glucose variability on prognosis using continuous glucose monitoring data, while remaining cautious regarding the potential adverse effects of insulin on beneficial metabolic pathways, such as fatty acid oxidation (White et al., 1987; Nadamuni et al., 2022; Collins and Huen, 2023).

### 4.2 Limitations of the study

Although PSM effectively balanced baseline characteristics (SMD < 0.1 after matching), this study has the following limitations:

First, despite rigorous PSM and multivariate adjustment, the observational design cannot fully exclude ‘confounding by indication.’ The decision to administer insulin may be a marker of greater illness severity not captured by the available acuity scores (e.g., SOFA, APACHE II). A related methodological concern is the potential inclusion of patients with undiagnosed diabetes. While we relied on documented international classification of diseases codes to ensure a non-diabetic cohort, the utility of HbA1c for confirmation was limited by a missing rate exceeding 90% in the MIMIC-IV database. Moreover, defining occult diabetes or prediabetes in the ICU setting using admission glucose is scientifically challenging, as acute stress hyperglycemia profoundly alters glycemic profiles. Therefore, the lack of specific insulin initiation criteria (e.g., glucose thresholds) and the potential for unmeasured baseline dysglycemia represent key limitations, and causality cannot be definitively inferred from this observational data (Schuetz et al., 2011; Wang et al., 2017; Costantini et al., 2021).

Second, data missingness remains a concern. The lack of data on insulin dosage, specific glycemic control targets, and glycemic fluctuation ranges prevents the analysis of dose-response associations or the assessment of the frequency and severity of hypoglycemic events. While hypoglycemia induced by insulin therapy may be associated with prolonged hospital stays, this association cannot be verified with the current dataset.

Third, there is inherent population heterogeneity. Although subgroup analysis showed consistent effects, there is an intrinsic difference between different sepsis subtypes (e.g., Gram-positive vs. Gram-negative infection). Given the emerging evidence that insulin differentially modulates biofilm formation in a microbe-specific manner (Plotkin et al., 2023), the inability to distinguish infection subtypes may obscure varying therapeutic responses and represents an important area for future investigation. Additionally, differences in treatment modalities (e.g., subcutaneous injection vs. infusion pump administration) could not be distinguished.

Fourth, the study is limited by its key outcome measures. The focus was solely on 28-day mortality, with a lack of endpoints such as organ function recovery time, long-term cognitive impairment, and quality of life. Given that insulin-like growth factor-binding protein-3 (IGFBP-3) is a strong predictor of long-term mortality in sepsis, future studies should incorporate this biomarker to improve risk assessment (Mearelli et al., 2025).

### 4.3 Future research directions

Based on the findings of this study, future research should prioritize addressing the following clinical and mechanistic issues:

First, at the mechanistic level, exploring the immunogenicity of insulin oxidation products and their differential activation of signaling pathways (PI3K/Akt, AMPK) may help explain the heterogeneity of therapeutic efficacy observed in clinical practice (Rena et al., 2017; Huang et al., 2018; Clemen et al., 2024). Furthermore, the role of insulin as an interkingdom signaling molecule requires deeper investigation; transcriptomic and metabolomic analyses could elucidate how specific insulin-to-glucose ratios alter bacterial virulence gene expression and host-pathogen interactions (Plotkin et al., 2023). Additionally, the role of epigenetic regulators, such as long non-coding RNA maternally expressed gene 3, in mediating insulin resistance should be elucidated via transcriptomics analysis (Pan and He, 2020; Wang C. et al., 2021). An immunosuppressive marker, the dynamic changes in IGFBP-3 may also serve as a valuable predictor of response to insulin treatment (Mearelli et al., 2025).

Regarding intervention strategies, the protective effect of insulin observed in this study requires validation through prospective randomized controlled trials (RCTs). Given that insulin increases the risk of hypoglycemia, novel glucose-dependent insulinotropic polypeptides, including glucagon-like peptide-1 receptor agonists (GLP-1RAs), may serve as safer alternative options. These agents regulate insulin secretion via a glucose-dependent mechanism and possess anti-inflammatory and organ-protective properties, potentially making them suitable for patients with sepsis complicated by hyperglycemia and organ dysfunction (Yang et al., 2021).

From a precision medicine perspective, metabolic intervention via ketogenic diets (KD) combined with insulin may synergistically improve mitochondrial function (Rahmel et al., 2024). Future efforts should focus on establishing an immune-metabolic phenotyping system for sepsis to clarify which subgroup, such as non-diabetic patients or those with high SOFA scores, benefits most from insulin therapy. Moreover, incorporating microbial insulin sensitivity testing, or “hormone biofilmgrams” as proposed by Plotkin et al., could eventually guide personalized insulin therapy based on the specific pathogen involved (Plotkin et al., 2023). Furthermore, the combined application of insulin with therapies regulating programmed cell death, including apoptosis, pyroptosis, and ferroptosis, represents a promising therapeutic direction (Wu et al., 2025).

## 5 Conclusion

In conclusion, this study demonstrates that insulin administration in non-diabetic patients with sepsis is associated with a significant reduction in 28-day mortality, independent of baseline confounding factors. However, increased ICU and hospital lengths of stay suggest that insulin may not shorten the overall duration of hospitalization. Future research should focus on elucidating the molecular mechanisms underlying insulin’s protective effects and exploring alternative metabolic interventions to optimize sepsis management.

## Data Availability

The original contributions presented in the study are included in the article/**Supplementary Material**. Further inquiries can be directed to the corresponding author.
