# Supplementary material for: The effect of insulin on 28-day mortality in non-diabetic sepsis patients: a retrospective study
Source: Front Cell Infect Microbiol. 2026 Apr 15;16:1806847. doi: 10.3389/fcimb.2026.1806847 (PMC13124701; doi:10.3389/fcimb.2026.1806847)
Supplement: Supplementary file 1 [file Table1.docx]

**Table. S1** Comparison of Last Glucose Measurements Between Insulin and Non-Insulin Groups

| Variable | Before PSM | |  | After PSM | |  |
| --- | --- | --- | --- | --- | --- | --- |
|  | Non-Insulin | Insulin | p-value | Non-Insulin | Insulin | p-value |
|  | 10117 | 1172 |  | 2178 | 782 |  |
| **Glucose**(mmol/L) | 105.00 (92.00, 125.00) | 111.00 (97.00, 140.00) | <0.001 | 105.00 (92.00, 125.00) | 112.00 (98.00, 143.00) | <0.001 |
